# New insights toward personalized therapies for vulvovaginal candidiasis and vaginal co-infections

**DOI:** 10.3389/fmicb.2025.1625952

**Published:** 2025-08-08

**Authors:** Francesco De Seta, Adilia Warris, Elena Roselletti

**Affiliations:** ^1^Department of Obstetrics and Gynaecology, IRCCS San Raffaele Scientific Institute, Milan, Italy; ^2^Department of Biosciences, Faculty of Health and Life Sciences, Medical Research Council Centre for Medical Mycology at the University of Exeter, Exeter, United Kingdom

**Keywords:** vulvovaginal candidiasis, vaginal co-infections, microbiota, lactic acid, immune cell dysfunction

## Abstract

Lower genital tract infections, particularly vulvovaginal candidiasis (VVC) and bacterial vaginosis (BV), are among the most prevalent infections in women worldwide, especially those of reproductive age. These conditions not only cause significant clinical symptoms but also severely impact women’s quality of life and mental health. Despite extensive research on the pathogens involved, substantial gaps remain in understanding the vaginal immune response and the complexity of the vaginal ecosystem, which is largely shaped by a *Lactobacillus*-dominated microbiota and the high concentration of lactic acid, contributing to the vagina’s unique acidic pH. This review explores the underlying pathophysiology of VVC, BV, and fungal-bacteria co-infections, as well as conventional and emerging treatments, including zinc, *Lactobacillus* spp., and lactic acid. The challenges of antifungal drug resistance are also discussed, in parallel with immune cell dysfunction and its potential link to the vaginal microbiota and ecosystem. Personalized treatments and approaches tailored to the individual vaginal environment are essential for maintaining eubiosis and preventing recurrent infections. Future research should prioritize modulating host and environmental factors rather than targeting pathogens alone, to develop targeted therapies that prevent reinfection, minimize side effects, reduce development of drug-resistance, and ultimately improve women’s health outcomes.

## 1 Introduction

Lower genital tract infections and sexually transmitted infections (STIs) are among the most common primary reasons for women to visit the gynecology clinic. These conditions include a wide range of clinical manifestations, including bacterial vaginosis (BV), vulvovaginal candidiasis (VVC), aerobic vaginitis (AV), and STIs caused by *Chlamydia*, *Neisseria gonorrhoea*, *Trichomonas*, and human immunodeficiency virus (HIV) (Pramanick et al., 2019). BV and VVC are the two most prevalent vaginal infections globally and are particularly common among women of reproductive age (Arastehfar et al., 2021; Denning et al., 2018; Sobel and Vempati, 2024). Understanding the complexity of these infections is crucial to addressing the widespread health challenges they pose. These infections significantly affect women’s quality of life and mental health (Neal and Martens, 2022), underscoring the need for a deeper exploration into their underlying mechanisms to improve the management of these infections. While substantial research has focused on pathogens, there remains a notable gap in our understanding of the human vagina’s physiological immune response. The unique acidic environment and diverse microbiota of the vagina further contribute to this complexity, with additional influences from factors such as ethnicity, socioeconomic status, and lifespan. To improve the treatment of VVC, in both its acute and recurrent forms, and its co-infections with anaerobic bacteria associated with BV, it is essential to investigate beyond the pathogens themselves. This requires examining the role of vaginal pH, lactic acid, and neutrophil (PMN) recruitment and dysfunction, as well as understanding how these factors influence the host’s immune response. Further research into how these elements interact in *in vitro* and *ex vivo* systems that mimic the human vaginal environment, along with studies involving human vaginal samples and patient-centered research, may lead to the development of novel, effective personalized therapies. Such advancements could ultimately improve women’s health outcomes by targeting individual vaginal ecosystems, preventing dysbiosis, and reducing infections.

## 2 Vulvovaginal candidiasis

Vulvovaginal candidiasis affects approximately 75% of women at least once in their lifetime, with 5–10% developing recurrent VVC (RVVC), defined as four or more symptomatic episodes per year, potentially persisting for decades (Donders et al., 2022; Denning et al., 2018; Neal and Martens, 2022; Rosati et al., 2020). Together, these infections account for half a billion cases annually (Roselletti et al., 2023). Predisposing factors for VVC include diabetes mellitus, contraceptive use, broad-spectrum antibiotics, pregnancy, and host genetics (Denning et al., 2018; Tsega and Mekonnen, 2019). Genetic mutations, such as polymorphisms in TLR2 and mannose-binding lectin 2, have been linked to RVVC, possibly increasing susceptibility to infection and leading to a hyperinflammatory response to *Candida* colonization and invasion (Rosati et al., 2020). However, no clear predisposing factors are identified in 20–30% of women with acute or recurrent infections (Rosati et al., 2020). Symptoms of VVC include irritation, itching, burning, redness, pain, dyspareunia, and vaginal discharge, which interfere with normal daily activities, and vary in severity among patients (Denning et al., 2018; Neal and Martens, 2022). Beyond physical discomfort, VVC and RVVC significantly reduce quality of life, leading to psychosocial issues such as increased stress, anxiety, depression, and social isolation (Neal and Martens, 2022). Acute and recurrent forms of VVC also globally contribute to a significant economic burden, affecting both healthcare systems and individuals. An annual treatment cost of at least $368 million has been estimated (Nyirjesy et al., 2022), with global losses amounting to approximately $14 billion due to a decline in productivity (Neal and Martens, 2022). The most common pathogen responsible for VVC is *Candida albicans*, but infections caused by non-*albicans Candida* (NAC) species are rising, particularly in developing countries where these infections range from 21 to 72%. The primary NAC species associated with VVC include *Candida glabrata*, *Candida krusei*, *Candida parapsilosis*, *Candida tropicalis, Candida dubliniensis, Candida lusitaniae*, and *Candida africana* (Pramanick et al., 2019; Arastehfar et al., 2021; Tsega and Mekonnen, 2019; Dunaiski et al., 2022; Monroy-Perez et al., 2016; Bitew and Abebaw, 2018; Mukasa et al., 2015; Brandolt et al., 2017; Waikhom et al., 2020; Khan et al., 2018; Fakhim et al., 2020). VVC infections caused by *C. albicans*, and NAC species have also increased in postmenopausal women, and are primarily due to an imbalance in the vaginal microbiome, rather than being estrogen related. During the childbearing age, estrogen promotes the microbial balance (eubiosis) and a low vaginal pH. In postmenopausal women, decreased or absent estrogen secretion leads to thinner, less elastic vaginal tissues, depletion of lactobacilli, and an increase in pH, resulting in higher susceptibility to infections (Donders et al., 2022; Al Halteet et al., 2020; Kim and Park, 2017). Postmenopausal women with diabetes or those undergoing hormone replacement therapy have the higher risk for complicated VVC (Kim and Park, 2017). In symptomatic *C. albicans*-induced VVC, the pathogen activates inflammasome receptors in vaginal epithelial cells through the production of virulence and immuno-inflammatory factors, triggering the release of cytokines and a neutrophilic inflammatory response, which often fails to resolve the infection (Roselletti et al., 2017; Roselletti et al., 2019b; Roselletti et al., 2019a; Roselletti et al., 2023; Cheng et al., 2024). The pathology of NAC species-induced VVC is currently poorly understood, however by contrast, tends to provoke less inflammation and PMN recruitment in *in vitro* and mouse models (Nash et al., 2016; Willems et al., 2018). Absence or significant reduction of PMN recruitment is also observed in vaginal samples from women with acute VVC caused by *C. glabrata* VVC (Roselletti et al., 2023).

## 3 Bacterial vaginosis and VVC co-infections

**Figure 1:**
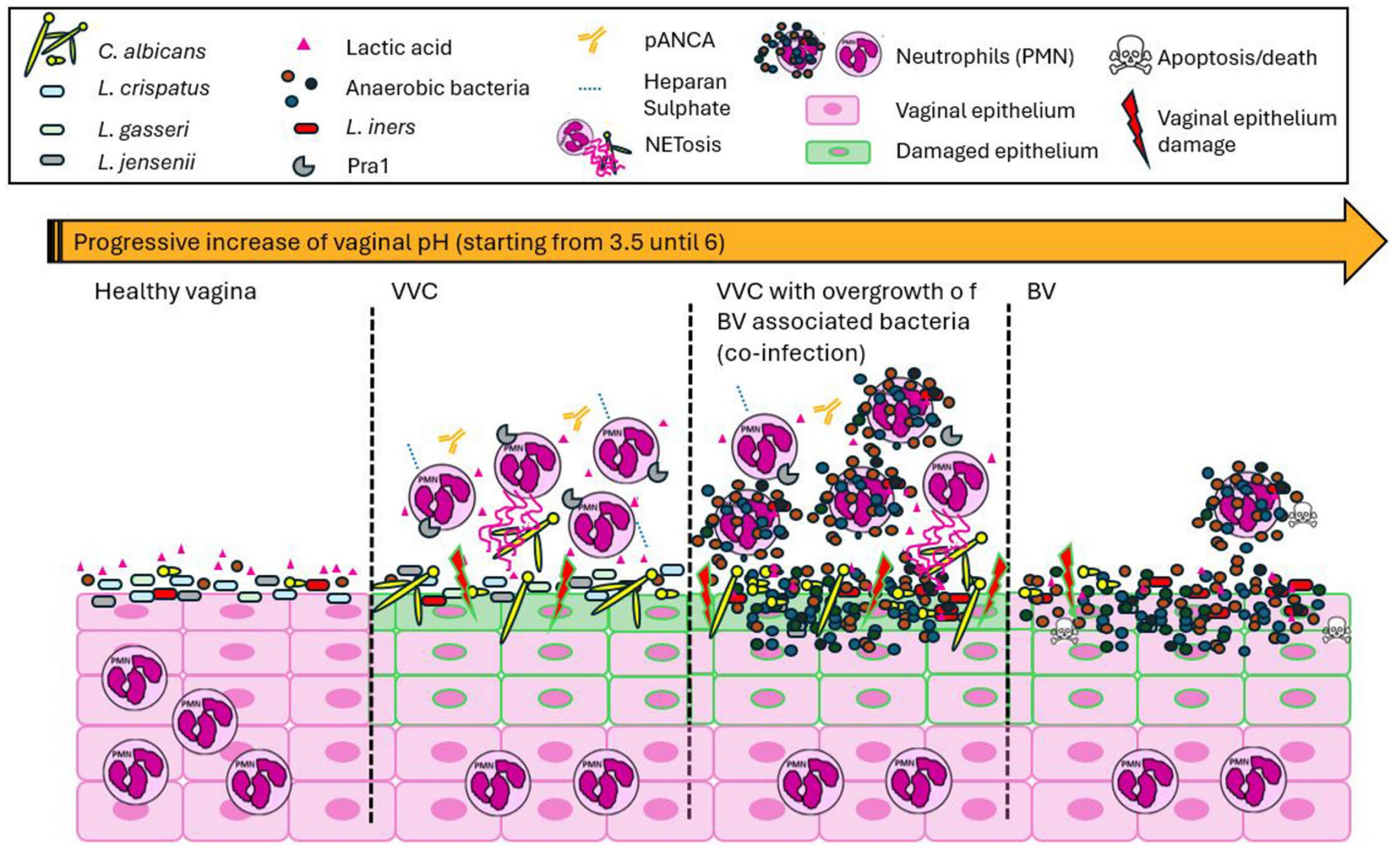
Distinct manifestations of vaginal infections. The healthy human vagina is characterized by a microbiota predominantly composed of *Lactobacillus* spp., which produce lactic acid and maintain an acidic environment, with absent host immune cell presence. Vaginal candidiasis (VVC) is characterized by a slight or no increase in acidic pH, an overgrowth of *Candida*, and a heightened host inflammatory response, primarily involving the recruitment of polymorphonuclear leukocytes (PMNs) that are unable to resolve the infection. This is mainly due to the involvement of proteins such as Pra1, pANCA antibodies, and heparan sulfate. During VVC, *Lactobacillus* spp. are still present, and no significant correlation with microbiome imbalance is observed. A decrease or complete absence of *Lactobacillus* spp. is associated with co-infections of *Candida* and anaerobic bacteria, as well as bacterial infections alone, inducing an increase in the vaginal pH. VVC, whether as a mono- or co-infection, is marked by an aberrant inflammatory response due to PMN accumulation, significant epithelial damage, and impaired infection resolution. In contrast, BV is characterized by the absence of PMN infiltration and the apoptosis of host cells.

Bacterial vaginosis is a common vaginal disorder more than an infection, and it is characterized by a shift from protective, hydrogen peroxide-producing *Lactobacillus* species to a polymicrobial community dominated by anaerobic bacteria, including *Gardnerella vaginalis*, *Atopobium vaginae*, *Prevotella*, and *Mobiluncus* species (Abou Chacra et al., 2021). The pathogenesis of BV remains incompletely understood, but key factors involve the disruption of the vaginal microbiome, and the subsequent loss of the acidic environment maintained by *Lactobacilli* (Chen et al., 2021). *Gardnerella vaginalis* plays a pivotal role in this process by forming biofilms on the vaginal epithelium, which provide a scaffold for the colonization of other anaerobes, and which are resistant to host immune responses and antibiotic treatments, contributing to the chronic and recurrent nature of BV (Chen et al., 2021; Onderdonk et al., 2016). Recent studies suggest that the host immune system plays a limited role in actively combating the microbial imbalance, contributing to the high recurrence rate following antibiotic treatment (Machado et al., 2015; Amabebe and Anumba, 2022). BV is often associated with specific symptoms such as fishy vaginal odor, thin grayish-white discharge, itching and irritation, although many women may be completely asymptomatic (Kairys et al., 2024). BV is also associated with an increased risk of STIs, preterm birth, and pelvic inflammatory disease, impacting women’s physical and mental health (Kairys et al., 2024). Addressing BV effectively requires considering both its physical and psychological effects. VVC and BV induce distinct clinical manifestations and pathologies, with VVC characterized by an aberrant inflammatory response and PMN accumulation and BV by a complete absence of PMN infiltration and epithelial apoptosis (Roselletti et al., 2023; Roselletti et al., 2019b; Roselletti et al., 2020). Despite these differences, 30% of VVC cases involve bacterial co-infections, most commonly with *G. vaginalis*, which increases clinical severity and complicates diagnosis and treatment (Sobel et al., 2013) (Figure 1). Interestingly, the predominant *Candida* species isolated during bacterial vaginal co-infection are *C. albicans* and *C. glabrata* (Pramanick et al., 2019). The co-occurrence of vaginal infections characterized by the overgrowth of *C. albicans* and *G. vaginalis* at the same time is completely understudied but poses significant therapeutic challenges, as antifungal treatments for VVC do not address the bacterial imbalance in BV, and vice versa (Sobel and Vempati, 2024). To find targets for intervention, it is important to fully characterize the underlying pathologies.

## 4 VVC treatment and antifungal drug resistance

Treatment of VVC and co-infections is critical, especially in pregnant women, as *Candida* colonization is linked to preterm birth, infant mortality, and neonatal invasive candidiasis (Tsega and Mekonnen, 2019). Co-infections with anaerobic bacteria, such as *G. vaginalis*, further increase the risk of pregnancy complications and STIs (Kairys et al., 2024). Treatment in pregnant women remains challenging due to the risks associated with long-term fluconazole use and frequent recurrences after discontinuation, highlighting the need for safer, more effective, therapies (Neal and Martens, 2022). Current treatment guidelines for acute VVC recommend various prescription and over-the-counter (OTC) agents, typically azoles, administered topically or orally. However, treatment of RVVC is more complex, and differ from country to country, depending on regulatory factors. The standard approach generally involves 7–14 days of induction therapy with an oral azole, followed by weekly maintenance treatment for 6 months (Donders et al., 2022). For NAC infections, boric acid is often recommended for the same duration (at least 7–14 consecutive days) (Neal and Martens, 2022; Centers for Disease Control and Prevention, 2021; Sobel et al., 2004). However, it is estimated that 50% of women with NAC infections are completely asymptomatic (Kennedy and Sobel, 2010). For this reason, the most recent guidelines recommend that long-term treatment be offered only to symptomatic women with no other identifiable cause, using not only boric acid but also nystatin or amphotericin B (Vieira-Baptista and Sobel, 2023). Despite these treatments, pharmacological control of VVC, RVVC, and co-infections, remains challenging due to antimicrobial therapy not eliminating the risk of re-infections and emergent antimicrobial resistance developing due to prolonged or repeated use of a single agent, mainly azoles (Agrawal et al., 2023; Marchaim et al., 2012; File et al., 2023). Symptom resolution is achieved in about 90% of women receiving RVVC maintenance therapy (Sobel et al., 2004), but more than 50% experience recurrence and re-infections after stopping treatment (Collins et al., 2020; Neal and Martens, 2022; Crouss et al., 2018). Vaginal co-infections show further significant therapeutic implications and complicate treatments. Up to 74% of women with recurrent BV have *Candida* colonization, leading to symptomatic co-infection in around 30% of cases (Sobel et al., 2013; Redondo-Lopez et al., 1990). Currently, it is not standard practice to treat both fungal and bacterial infections simultaneously (Sobel et al., 2013; Balkus et al., 2011), leading to a cycle of recurrent VVC following BV treatment (Sobel and Vempati, 2024). This phenomenon, although not officially recognized in all medical guidelines, is well-known among patients and health care practitioners. As a result, many women with recurrent BV often self-use oral fluconazole for prophylaxis during symptomatic BV episodes (Sobel and Vempati, 2024). This unofficial practice contributes to the development of fluconazole resistance in *C. albicans* isolates, leading to refractory VVC episodes (Sobel and Vempati, 2024). Fluconazole-resistant *C. albicans* and azole-resistant NAC species are becoming increasingly problematic due to the indiscriminate use of antifungals. Despite progress in understanding azole resistance, effective solutions remain limited. Approximately 7.3% of RVVC cases are associated with clinically defined fluconazole-resistant *C. albicans* (File et al., 2023). Personalized therapy, taken into account both the role of fungal and bacterial pathogens, tailored to each woman’s medical history and resident *Candida* species, could help prevent reinfection, minimize side effects, and reduce the risk of drug-resistant pathogens. Drug resistance is particularly prevalent in developing countries, where the diagnosis of vaginal infections is often made on clinical symptoms rather than laboratory testing, and NAC species are a more common cause of VVC (Tsega and Mekonnen, 2019; Bitew and Abebaw, 2018; Mukasa et al., 2015; Arastehfar et al., 2021; Brandolt et al., 2017; Waikhom et al., 2020; Khan et al., 2018; Guzel et al., 2013). *C. glabrata* and *C. krusei*, the two most isolated NAC species, naturally resistant to fluconazole, are increasingly replacing *C. albicans* in these regions (Bitew and Abebaw, 2018). Treating azole-resistant vaginitis remains a clinical challenge. Boric acid, nystatin, amphotericin B, and flucytosine are possible alternatives for azole-resistant RVVC, but NAC infections are more difficult to manage due to their inherent or acquired resistance to common antifungals (Neal and Martens, 2022). Amphotericin B, although effective, is not routinely prescribed due to its high cost, difficulty of administration, and significant kidney toxicity (Tsega and Mekonnen, 2019). Similarly, flucytosine remains effective against many *Candida* species, but it is also prohibitively expensive in many developing countries, and monotherapy is associated with rapid resistance development (Paavonen and Robert, 2020).

## 5 Preventive measures

Several strategies to develop a vaccine against *Candida* infections were tested, including inactivated yeast and protein-based formulations, trying to consider what type of immune response is protective under natural conditions (Vecchiarelli et al., 2012). Despite limited early success, recent advancements have renewed interest, with several peptide-based and DNA vaccines targeting specific *Candida* antigens in development. Among these, the immunotherapeutic NDV-3A vaccine has shown promise. It contains a recombinant *C. albicans* adhesin/invasin protein and utilizes an innovative delivery system to enhance immunogenicity. In an exploratory phase 2, randomized, double-blind, placebo-controlled trial conducted on 188 women with recurrent vulvovaginal candidiasis (RVVC), aged 18–55 years, who were using an approved method of birth control and presented with a clinically diagnosed active episode of VVC, NDV-3A was demonstrated to elicit a targeted immune response, significantly reducing the incidence and recurrence of VVC (Edwards et al., 2018). Specifically, the study showed that a single intramuscular dose of NDV-3A was safe, generated rapid and robust B- and T-cell immune responses, and reduced the incidence and frequency of symptomatic RVVC episodes for up to 12 months in women under 40 years of age (Edwards et al., 2018). This vaccine represents a transformative step toward a preventative strategy that could greatly improve the quality of life for those prone to VVC. Moreover, it also serves as an important tool for the prevention of disseminated candidiasis in neonates (Singh et al., 2022).

## 6 The vaginal microbiota

**Figure 2:**
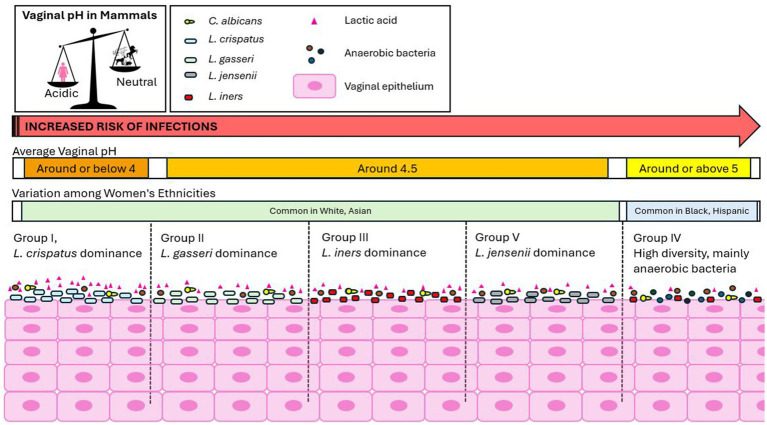
The unique human vaginal environment. The human vagina is characterized by a microbiota predominantly composed of *Lactobacillus* species, which helps maintain an acidic vaginal pH, a unique feature among mammals. Five major groups of vaginal microbial communities have been identified: Groups I, II, III, and V: These groups are characterized by lower microbial diversity and are predominantly dominated by *Lactobacillus* spp. Group IV: This group exhibits greater microbial diversity, with a lack of *Lactobacillus* dominance and a higher prevalence of anaerobic bacteria, which increases the risk of infections. Groups I, II, III, and V are more commonly found in white and Asian women, while Group IV is predominantly observed in Black and Hispanic women. The physiological vaginal pH varies slightly depending on the microbial community group. Women in Group I tend to have the lowest pH (around or below 4.0), while Group IV has the highest pH (around 5.4). The pH values for Groups II, III, and V fall in between.

The human vagina is a unique environment, characterized by a microbiota predominantly composed of *Lactobacillus* spp., accounting for approximately 80% of the total microbial population (Pramanick et al., 2019). The vaginal pH is acidic, ranging between 3.5 and 5, a unique feature among mammals (Miller et al., 2016; Pramanick et al., 2019) (Figure 2). Five major groups of vaginal microbial communities have been identified, each varying in the type and number of *Lactobacillus* spp., bacteria, archaea, viruses, and fungi. Groups I, II, III, and V are characterized by lower microbial diversity, and are dominated by *Lactobacillus* spp.: *Lactobacillus crispatus* (Group I), *L. gasseri* (Group II), *L. iners* (Group III), and *L. jensenii* (Group V). Group IV, however, is characterized by greater diversity, with a lack of *Lactobacillus* dominance and a higher prevalence of anaerobic bacteria, increasing the risk of infections (Sun et al., 2023; Ottinger et al., 2024; Ravel et al., 2011; Wei et al., 2024). Groups I, II, III, and V are more common in white and Asian women, while Group IV is predominantly found in Black and Hispanic women (Petrova et al., 2015) (Figure 2). Despite the identification of these five main groups, the vaginal microbiome is highly variable across individuals due to factors such as sexual habits, menstrual hygiene, stress, geography, and socioeconomic status (Sun et al., 2023). Additionally, the microbiome composition changes throughout different life stages, including puberty, pregnancy, postpartum, and menopause (Ottinger et al., 2024).

## 7 Vaginal pH and lactic acid

The physiological vaginal pH varies slightly depending on the microbial community group. Women in Group I have the lowest pH (around or below 4.0), while Group IV has the highest (around 5.4). Groups II, III, and V fall in between, with pH values of around 4.7, 4.2, and 4.8, respectively (Ravel et al., 2011; Aldunate et al., 2013). In Group IV, the higher pH is due to the decreased presence of *Lactobacillus* spp., with *L. iners* being the only exception (Aldunate et al., 2013) (Figure 2). The acidic vaginal pH is primarily due to lactobacilli metabolites, with lactic acid being one of the most significant. Lactic acid, a lipid-soluble, membrane-permeable carboxylic acid, exists in two forms based on the environmental pH: a neutral protonated form under acidic conditions and a charged, unprotonated lactate anion under neutral conditions (Aldunate et al., 2013). It is the protonated form that provides protective activity for the host (Aldunate et al., 2013). Physiological vaginal lactic acid concentrations range from 55 to 111 mM, and it is constituted by racemic DL-lactic acid isoforms, with higher concentrations as the pH becomes more acidic. Human metabolism produces only the L-isomer, with less than 15% of the total vaginal lactic acid coming from vaginal epithelial metabolism (Aldunate et al., 2013), while *Lactobacillus* spp. produce different isomers: *L. crispatus and L. gasseri* produce both D- and L-lactic acids, *L. iners* produces only the L-isomer, and *L. jensenii* produces only the D-isomer (Tachedjian et al., 2017). The benefits of *Lactobacillus* spp. extend beyond lactic acid production and pH regulation, including the production of hydrogen peroxide, bacteriocins, competitive adherence to the vaginal epithelium, and immunomodulation (Mejia et al., 2023). In contrast to VVC, which typically involves a single pathogen inducing infection, BV represents a severe polymicrobial dysbiosis with the loss of protective *Lactobacillus* species and an overgrowth of anaerobic bacteria (Sobel and Vempati, 2024). In VVC, changes in the vaginal microbiota are associated with an increased pro-inflammatory response in vaginal epithelial cells due to surface exposure and invasion by the fungus. In cases of VVC co-infections, this response is compounded by the severe polymicrobial dysbiosis of BV, leading to increased inflammation and symptom severity (Sobel and Vempati, 2024). Vaginal microbial perturbations are associated with adverse outcomes such as preterm birth, pelvic inflammatory disease, urinary tract infections, and STIs (Mejia et al., 2023), similar to the complications seen in VVC and BV. This suggests a connection between various vaginal infections and microbiome composition. Changes in the microbiome not only affect pH but may also influence the progression to more serious conditions and infections. Lactic acid, with an acid dissociation constant of solution (pKa) of 3.9, is significantly more concentrated in the vagina than in other parts of the body (Foucher and Tubben, 2023; Aldunate et al., 2013). The vaginal distinct acidity raises concerns about the limitations of current cell line and mouse models for studying vaginal infections, as these models typically have a neutral vaginal pH (Roselletti et al., 2019a). To more accurately reflect the human vaginal environment, adjustments to model systems are needed. Further research is needed to understand how lactic acid concentrations and isomers vary across different microbial communities over the lifespan, and whether targeting its modulation could help maintain a balanced vaginal microbiome, preventing the progression to immunopathology, inflammatory diseases, or infections.

## 8 The dual role of lactic acid

Lactic acid has well-documented protective effects against pathogens. At vaginal physiological concentrations, it shows potent virucidal activity against HIV (Aldunate et al., 2013; Tachedjian et al., 2017) and inhibitory effects against Herpes Simplex virus types 1 and 2 (HSV-1 and HSV-2) (Tachedjian et al., 2017), and *Chlamydia trachomatis* (Edwards et al., 2019). It also protects against BV-associated bacteria such as *Escherichia coli*, *G. vaginalis*, *Neisseria gonorrhoeae*, and Group B *Streptococcus* (Plummer et al., 2021; Tachedjian et al., 2017). Notably, lactic acid’s protective effects are distinct from its acidity alone and surpass those of acetic acid (Tachedjian et al., 2017; Aldunate et al., 2013). However, several studies have not found a correlation between a protective effect of lactic acid or *Lactobacilli* against *Candida* species in VVC (Tachedjian et al., 2017). This may be explained by the fact that yeast are highly tolerant to low pH levels (Lourenco et al., 2018). The impact of lactic acid on the host remains controversial, and still not fully understood. In the vagina, *Lactobacilli* are associated with a non-inflammatory environment (Tachedjian et al., 2017; Sakai et al., 2004; Nikolaitchouk et al., 2008; Kyongo et al., 2012), while dysbiosis and infections are associated with inflammation and a higher risk of STIs (Tachedjian et al., 2017). Some studies suggest lactic acid may have pro-inflammatory effects on immune and vaginal epithelial cells (Witkin et al., 2011; Mossop et al., 2011). Additionally, acidic pH has been shown to reduce the motility and viability of immune cells like monocytes, macrophages, and lymphocytes potentially compromising their role in preventing HIV transmission (Olmsted et al., 2005). Although this study did not specifically examine lactic acid, it raises important questions about its effects, particularly in combination with acidic pH, on immune cell function in the vaginal environment, suggesting that its role may not always be protective. Further research is needed to clarify the role of lactic acid in the host and explore its therapeutic potential in combination with other molecules or its involvement in vaginal infections.

## 9 *Lactobacillus* spp. and lactic acid as treatments for vaginal infections

*Lactobacillus* spp. and lactic acid play vital roles in maintaining vaginal health and have been explored as treatments for infections like BV and VVC (Abavisani et al., 2024). Lactic acid’s key strength lies in its ability to restore and maintain the acidic vaginal pH and inhibiting pathogen growth (Barrientos-Duran et al., 2020). Research shows that lactic acid, in gel or suppository form, is particularly effective in treating BV when used with antibiotics, helping to restore the microbiome and reduce symptoms (Plummer et al., 2021; Abbasi et al., 2022). It has also shown promise in prophylactic use, helping to prevent BV and VVC recurrences by maintaining a stable vaginal environment (Werner et al., 2022). However, lactic acid alone is often insufficient and may require combination with other treatments for optimal efficacy (Werner et al., 2022). *Lactobacillus* spp., particularly *L. crispatus* and *L. jensenii*, dominate a healthy vaginal microbiome and are crucial for lactic acid production. Probiotics containing *Lactobacillus* strains can help re-establish a balanced vaginal microbiome and reduce harmful pathogen overgrowth (Liu et al., 2023; Cribby et al., 2009). Probiotics are effective in treating BV and may reduce recurrences when used with antibiotics (Chen et al., 2022). For VVC, they can be, but are not always, associated with antifungal effects, stabilizing the microbiome and preventing *Candida* overgrowth, although the evidence is less robust (Akinosoglou et al., 2024; Gaziano et al., 2020; van de Wijgert and Verwijs, 2020). However, the use of *Lactobacillus* probiotics has several limitations. While generally beneficial, probiotics can sometimes disrupt the vaginal microbiome, leading to imbalances or even irritation. Introducing external bacteria, such as *L. crispatus*, may disturb the natural balance of the vaginal ecosystem, potentially causing dysbiosis or overgrowth of beneficial bacteria, which can outcompete other essential microbes. The vaginal microbiome varies significantly between individuals, meaning what works as a beneficial probiotic for one woman might have adverse effects for another (Pagar et al., 2024; Chee et al., 2020). Moreover, the probiotic market is poorly regulated, leading to inconsistencies in product quality, concentration, and viability (van de Wijgert and Verwijs, 2020) and may result in side effects such as irritation or allergic reactions. Probiotic formulations also often contain additional components, like adjuvants, that can cause sensitivities or discomfort in some women. Additionally, the effects of probiotics are typically temporary (van de Wijgert and Verwijs, 2020), necessitating continuous use for sustained benefits, which may not always be practical or beneficial (Liu et al., 2023). Considering these aspects, both lactic acid and *Lactobacillus* probiotics could be promising treatments, but only when used as part of a broader, personalized strategy carefully tailored to each woman’s unique vaginal microbiome and specific infection.

## 10 The immunomodulatory role of zinc

**Figure 3:**
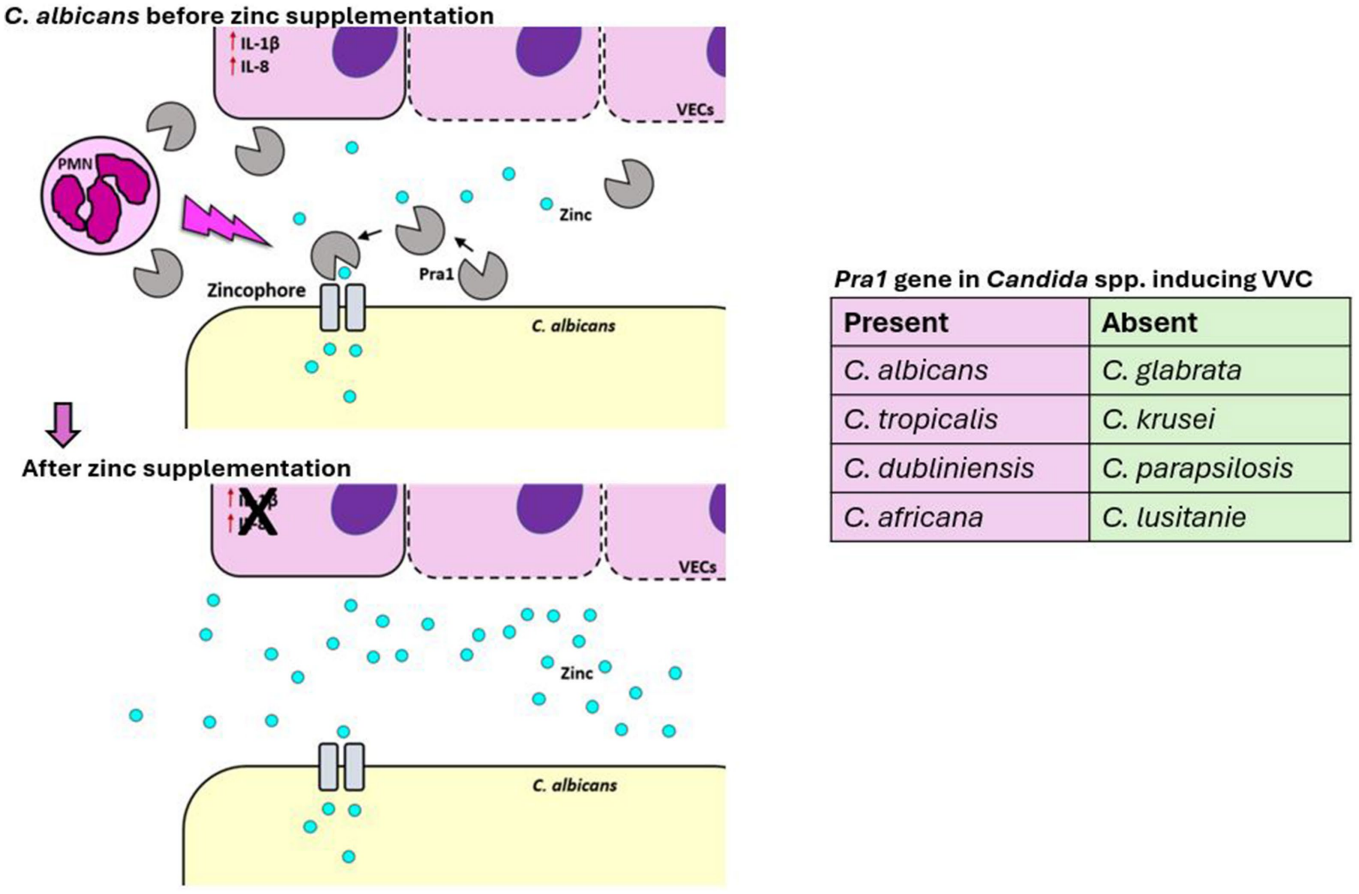
Effect of zinc supplementation on *C. albicans* during VVC. During VVC infection, *C. albicans* expresses and releases a zinc-binding protein, Pra1, which is linked to local inflammation and PMN recruitment, driving the VVC immunopathology. Since Pra1 facilitates zinc capture by the yeast, its expression is negatively regulated by zinc availability. Zinc supplementation effectively inhibits Pra1 expression, reducing PMN infiltration, inflammation, and reinfection in humans. From a therapeutic perspective, Pra1 is an attractive target for treating *C. albicans*-induced VVC, potentially improving the quality of life for affected women.

Our own research has introduced zinc supplementation as a promising therapeutic strategy for *C. albicans*-induced VVC (Roselletti et al., 2023). During infection, *C. albicans* expresses a zinc-binding protein, Pra1, which helps the yeast capture zinc from its environment, particularly when the nutrient is limited (Citiulo et al., 2012), as it is in the vaginal mucosa (Roselletti et al., 2023). Pra1 expression is linked to local inflammation and PMN recruitment, which drives the immunopathology of VVC. Because Pra1 is used by the yeast to capture zinc, its expression is negatively regulated by this micronutrient. Zinc supplementation effectively inhibits Pra1 expression, reducing PMN infiltration and inflammation in both *in vitro* and *in vivo* models at neutral and acidic pH (Roselletti et al., 2023) (Figure 3). In a pilot study, five out of six women who applied a zinc-containing vaginal gel for 3 months did not experience recurrence (Roselletti et al., 2023). From a therapeutic perspective, Pra1 is an attractive target for treating *C. albicans*-induced VVC, offering significant improvements in quality of life for affected women. However, many NAC species, such as *C. glabrata* and *C. krusei*, have lost the PRA1 gene during evolution (Roselletti et al., 2023) (Figure 3), limiting the efficacy of zinc treatment in such cases and posing a significant challenge for future treatment. Moreover, zinc may not be a viable option for fungal-bacterial vaginal co-infections due to its ineffectiveness against bacteria (Roselletti et al., 2023), as PRA1 gene, is unique to the fungal kingdom. These observations underline the critical need to find new targets for intervention in VVC infections, suggesting the possibility of directly targeting the host inflammatory response to treat VVC while reducing antifungal usage.

## 11 Immune cell dysfunction in VVC

While it is known that Pra1 and other immuno-inflammatory molecules play a role in PMN recruitment during VVC (Roselletti et al., 2023; Gabrielli et al., 2016; Richardson et al., 2018), why the PMNs are not able to clear the infection is unclear (Cheng et al., 2024; Kalia et al., 2019; Ardizzoni et al., 2021). One hypothesis is that the vaginal environment in VVC induces neutrophil dysfunction (Cheng et al., 2024). Heparan sulfate, a proteoglycan found on mammalian cell surfaces (Sarrazin et al., 2011), has been identified as a key contributor to this dysfunction, binding to CD11b on PMNs and impairing reactive oxygen species (ROS) production and NETosis, thereby reducing *C. albicans* clearance (Yano et al., 2017; Yano et al., 2014). estrogen may exacerbate this process by increasing heparan sulfate expression, and it has been shown that heparinase III can restore neutrophil activity in VVC-susceptible mice (Yano et al., 2017). Additionally, neutrophil hyperactivation, triggered by fungal virulence factors such as Pra1, candidalysin, and Secreted aspartyl proteinases (SAPs), can cause tissue damage through excessive NET formation, protease release, and ROS production (Roselletti et al., 2019a; Roselletti et al., 2023; Cheng et al., 2024). While these responses are meant to neutralize *C. albicans*, their premature release may result in tissue injury rather than effective pathogen clearance. This hyperactivation, compounded by the presence of markers like perinuclear anti-neutrophil cytoplasmic antibodies (pANCA), contributes to the inflammatory damage observed in VVC, suggesting a misalignment in the timing and location of neutrophil responses, worsening the infection (Cheng et al., 2024; Ardizzoni et al., 2020). Despite these insights, more research is needed to fully understand the role of neutrophil dysfunction in the pathophysiology of human VVC. Much of the current knowledge on VVC pathology is based on studies using cell lines and mouse models at neutral pH. While informative, these models do not accurately reflect the acidic vaginal environment characteristic of human VVC, which is shaped by a unique concentration of lactic acid (Roselletti et al., 2019a; Roselletti et al., 2023). PMNs are essential and effective immune cells in systemic candidiasis and other mucosal infections, such as oral candidiasis. However, they fail to provide protection in the vaginal environment, suggesting that this unique and specific setting plays a primary role in their impaired function.

## 12 Discussion and future implications

Acute and recurrent VVC infections are the most common vaginal infections globally, particularly among reproductive-age women. These conditions severely impact women’s quality of life, contributing to significant psychosocial and economic burdens across all income regions. Despite extensive research into the pathogens involved, the physiological immune response of the human vagina remains poorly understood and underexplored. This gap in knowledge can be attributed, in part, to the unique acidic vaginal environment, the extremely high physiological levels of lactic acid, and its highly diverse microbiome, influenced by factors such as ethnicity, socioeconomic status, and age. To advance our understanding, it is essential to study the effects of the acidic vaginal environment on host immune cells using models that closely mimic the human vaginal environment. These studies should include not only Lactobacilli and lactic acid but also local factors like estrogen, bacteria, mucus, and released metabolites. A deeper understanding of these elements could clarify critical targets for developing personalized therapies. Such strategies would be tailored to each woman’s unique vaginal microbiome and specific infection profiles, aiming to support individual vaginal ecosystems, prevent dysbiosis, and improve health outcomes by addressing imbalances before they result in infection.

## Author disclaimer

The views expressed are those of the author(s) and not necessarily those of the NIHR or the Department of Health and Social Care. For the purpose of open access, the author has applied a Creative Commons Attribution (CC BY) licence to any Author Accepted Manuscript version arising from this submission.
